# Suitability of memory aids and strategies for people with posterior cortical atrophy: protocol for a scoping review

**DOI:** 10.1186/s13643-023-02187-4

**Published:** 2023-03-30

**Authors:** A. Burbaite, S. Leeworthy, L. Hirst, E. Mioshi, L. Clare, S. Ahmed

**Affiliations:** 1 Department of Psychiatry, University of Oxford, Oxford, UK; 2 Department of Paediatric Neuropsychology, University Hospitals Bristol and Weston NHS Trust, Bristol, UK; 3 Academy Library, Royal United Hospitals, Bath, UK; 4 School of Health Sciences, University of East Anglia, Norwich, UK; 5 Centre for Research in Ageing and Cognitive Health, University of Exeter, Exeter, UK; 6 School of Psychology and Clinical Language Sciences, University of Reading, Reading, UK; 7 Nuffield Department of Clinical Neurosciences, University of Oxford, Oxford, UK

**Keywords:** Posterior cortical atrophy, Alzheimer’s disease, Memory, Memory aids, Memory strategies

## Abstract

**Background:**

Posterior cortical atrophy (PCA) is a neurodegenerative syndrome characterised by progressive visuospatial and visuoperceptual impairment. Recent research shows that memory impairment can also occur as an early symptom of the condition and that the impairment can be ameliorated by providing support in the memory recall phase, for example, by presenting a related cue. In Alzheimer’s disease (AD), which is defined by an amnestic syndrome, memory aids and strategies have been used to help support everyday memory, which in turn can have a positive impact on patient and carer outcomes. Similar support for PCA could be achieved by using memory aids and strategies which help to encode and/or retrieve information, yet there are currently no guidelines for memory strategies that may be suitable in PCA. Due to the central visual disorder that defines PCA, careful consideration is needed when making recommendations.

**Methods:**

A scoping review will be conducted of published studies that have assessed memory aids and strategies in people with AD and related dementias where memory is considered a core or supplementary feature, with the aim of distinguishing those that may be suitable or adaptable for PCA. The systematic search will include the electronic databases MEDLINE, PsycINFO and CINAHL, using search terms for dementia and memory aids and strategies identified in pilot searches. Findings will be mapped and described based on methods used, population, clinical data and memory aids and strategies identified.

**Discussion:**

The scoping review will give an overview of the memory aids and strategies used in people with AD and related dementias and identify characteristics, modality and pragmatics to evaluate their suitability and adaptability for a PCA population. Tailored memory support strategies for people living with PCA could improve memory performance, with knock-on positive effects on patient and carer outcomes.

**Supplementary Information:**

The online version contains supplementary material available at 10.1186/s13643-023-02187-4.

## Background

Posterior cortical atrophy (PCA) is a neurodegenerative syndrome defined by a progressive visuospatial and visuoperceptual impairment that cannot be attributed to ocular disease [1–3]. People with PCA experience impaired spatial perception, spatial awareness, orientation [3] and poor capacity for visual imagery [4]. The most common underlying cause in the majority of cases is Alzheimer’s disease (AD) [3, 5]. Although visuospatial deficits are the salient and defining feature of PCA, a recent consensus report has drawn attention to several additional cognitive symptoms that can often be detected early in the disease course [3]. Amongst these is a common complaint of declining memory, reported by up to two-thirds of patients [3]. Other surveys suggest that a similar number of patients experience some form of lapse in memory that interferes with their independence in daily activity [6, 7]. The nature of the memory impairment differs from the typical dense amnesia that is observed in AD. Instead, people with PCA have difficulty retrieving information that was previously learnt [7, 8], and evidence suggests that this may be due to disrupted attention to the information at the time of learning [7, 9]. Critically, if support is given at the time of memory retrieval, such as by providing a related cue, memory impairment can be ameliorated to achieve a high recall of information, similar to healthy participants. Evidence therefore suggests that due to the attentional underpinnings of memory impairment, people with PCA are able to derive greater benefit from strategies aiming to help direct attention and minimise distractions [8].

At the time of writing, there are no tailored approaches to support memory functioning in PCA, despite the evidence for a significant subset of patients experiencing these symptoms and the indication that memory could be well supported. The detrimental effect of memory impairment on a person’s life is well documented in other dementia syndromes [10, 11], associated with greater difficulties in performing activities of daily living [12–14], increased caregiver burden [15] and mood disorders such as depression [16]. Only one study has explored the real-world impact of memory impairment for people with PCA, revealing a direct link with low mood and apathy [17] which are, in turn, related to poorer long-term outcomes [18–21]. Extrapolating from the memory support literature in related dementia syndromes, we can assume that tailored strategies that meet the needs of a PCA population would have a broad-ranging positive impact for patients and carers. In people with AD where memory impairment is the salient feature, memory strategies and aids have an important assistive purpose by supporting function in everyday life. For example, memory strategies have been shown to have a positive impact on practical information learning, such as the names of people and household objects [22–25] and recall of recent events [26]. Electronic memory aids, such as smartphones, have demonstrated the potential to effectively enhance prospective memory by, for example, aiding with remembering appointments [27]. In both people with dementia and their carers, memory interventions have the potential to yield significant improvements in quality of life [28, 29].

The development of memory support that is accessible to people with PCA requires careful consideration due to the central visual disorder. This certainly presents challenges but we would argue that tailored memory strategies for this population should be considered a priority. Evidence from other dementia syndromes shows that compounded deficits lead to greater disability. For example, Parkinson’s disease is defined as a primary motor disorder characterised by tremor, but it is estimated that significant secondary cognitive impairment develops in 24–31% of cases [30]. The combination of motor and cognitive impairment leads to greater disability, poorer wellbeing and poorer long-term outcomes, compared to those with isolated motor problems [31, 32]. Similarly, in PCA, deficits in the visual domain compounded by even mild memory problems may contribute to increased disease burden [17]. We propose that memory aids will provide benefit, particularly in mild to moderate stages of disease. The aim of this review is not, therefore, to simplify the complex constellation of symptoms, but to share evidence-based post-diagnostic support strategies and identify fundamental amendments that may be needed to make these strategies accessible for people with PCA.

Memory interventions have been variably labelled. They can be broadly distinguished as “memory aids and strategies”, which can be introduced and used singly and may in some cases be chosen by the person or family without professional support, and “memory interventions”, which may utilise aids and strategies as part of an overall plan and are usually delivered by a professional in individual or group sessions (for example, memory support groups or cognitive rehabilitation programmes). The focus of this review will be on “memory aids and strategies”, defined as “devices or strategies that are deliberately used to enhance memory” [33]. There are two main categories of memory aids and strategies—internal and external [34, 35]. Internal strategies help to encode and retrieve information and include the use of mental images, using mnemonics (e.g. creating acronyms) and repeating information to oneself [34, 35]. External strategies and aids support memory function by manipulating or relying on the environment, such as using calendars, making lists, writing notes and trusting others to provide reminders [34, 35]. The use of technology to deliver memory aids and strategies (both internal and external) is a rapidly evolving sphere of research, already offering assistive technologies (AT) such as electronic medicine boxes, mobile tracking devices and smart phone applications [36, 37].

We will use the term “memory aids and strategies” as a broad term that includes both external and internal aids and strategies. This description permits any type of memory aid or strategy that helps people to encode and retrieve information.

The overall aim of this study is to conduct a scoping review to firstly identify memory aids and strategies used in AD and related dementias where memory is considered a core or supplementary symptom. Secondly, we will classify the characteristics, modality and pragmatics of the aids and strategies for use in a visually impaired population. Thirdly, we will appraise their suitability and adaptability for a PCA population. The majority of recent reviews on memory aids and strategies have not provided information on modality and none has focused on PCA. As such, this review will provide key information to highlight gaps in the evidence base and guide further research into cognitive support strategies for people with PCA. A scoping method was chosen in order to conduct the search in a rigorous and systematic approach, whilst also allowing the flexibility to include quantitative and qualitative studies.

## Methods/design

### Objectives

The core questions posed by this scoping review are “Which memory aids and strategies are used in AD and related dementias, what are the characteristics and modality (visual, verbal, auditory) of each, and what modifications are needed to make them accessible to people with PCA?”.

The literature will be systematically scoped following the framework outlined by Peters et al. [38], based on the original framework proposed by Arksey and O’Malley [39]. Additionally, we will follow PRISMA-ScR guidelines for scoping review publication [40]. Mapping of the literature will allow a summary and description of current memory strategies used, whilst seeking to highlight existing or adaptable resources that may be transferable to individuals with PCA.

### Search strategy

A search of relevant peer-reviewed, published literature will be made using the NICE Healthcare Databases Advanced Search to extract articles from the following electronic databases: MEDLINE, PsychINFO and CINAHL. The following search strategy will be applied: (dementia OR *DEMENTIA/ OR alzheimer* OR * “ALZHEIMER’S DISEASE”/ OR “posterior cortical atrophy” OR PCA OR “mild cognitive impairment”) AND (memory OR memories OR *MEMORY/) AND (“memory strateg*” OR “memory aid*” OR compensatory OR restorative OR “enhanced learning” OR external ADJ2 strateg* OR internal ADJ2 strateg* OR internal ADJ2 aid* OR external ADJ2 aid* OR “assistive technolog*” OR “assistive device*” OR “task modificat*” OR “task adaptat*” OR “environment* modificat*” OR “environment* adaptat*” OR remind* OR Plann* OR calendar* OR sign* OR list* OR checklist* OR diar* OR mental picture* OR “expand* rehearsal*” OR “space* retrieval*” OR cue* OR prompt* OR chunking OR “method of loci” OR mnemonic* OR “action learn*” OR “action based learn*” OR “errorless learn*” OR chaining OR modelling OR modeling OR “semantic associat*” OR “memory navigat*” OR “memory device*” OR “adaptive technolog*” OR “adaptive device*” OR “prompting tool*” OR “prompting device*” OR “electronic* device*” OR “electronic* aid*” OR smartphone* OR tablet* OR app*), with limited date range (30 years) and language restriction (English language only) (see attached search strategy in Additional file 1). The search strategy was developed for use by identification of key words from relevant articles, pilot searches and in consultation with experienced researchers and librarians. Articles published within 30 years of the present year were included, as pilot searches suggested low relevance of studies matching our criteria prior to this date. Articles will be restricted to those that have been published in English. The research team does not have the breadth of language proficiency needed to include papers published in all other languages, nor does the team have access to the resources to translate papers. Revised intervention terms do not include terms describing therapy or support group programmes delivered by a professional, as the specific aim of the study is to identify aids or strategies that are patient-led or patient-led with carer support and not those requiring group delivery or instructors. Additional search terms were included to capture digital aids, such as assistive technology as this is a novel and fast-evolving area [36].

Authors AB and SL will independently screen the titles and abstracts. Duplicate and irrelevant articles will be removed. The remaining full-text versions will be screened to ascertain whether they meet the pre-decided eligibility criteria for inclusion to produce the final list of articles to be included in the review. Any disagreements will be discussed and, if required, resolved by consultation with a senior researcher (SA). Where there is a conflict of interest, for example where a member of the review team is an author on a considered article, that team member will be excluded from the decision-making process for the articles in question.

### Study selection

#### Population

Studies that focus on memory aids and strategies (or equivalent term) in people with a clinical diagnosis of dementia will be included, inclusive of any type of dementia or severity. People with a clinical diagnosis of mild cognitive impairment (MCI) will also be included, where the patient cohort is defined by clinical criteria for MCI denoting preclinical dementia [41, 42, 43]. If studies are reporting on mixed populations, they will be included if at least 50% of the population has a relevant clinical diagnosis and if this data was separately identified. Studies will be included from community, outpatient, in-patient and residential settings, where a diagnosis has already been given and memory impairment has been profiled. There will be no limitations on age, gender or ethnicity.

#### Concepts

Studies that investigate memory aids or strategies (or equivalent term) as a primary or secondary intervention of interest, using internal or external methods, will be included. Studies using traditional and digital approaches will be included.

#### Context

Study selection will be restricted to articles published in English and where full text is available. Sources of information can include any existing peer-reviewed literature, including quantitative, qualitative or mixed method primary research studies, meta-analyses, systematic reviews and conference reports.

#### Exclusion criteria

- Non-dementia diagnosis
- Studies that did not measure memory strategies or aids
- Pharmacological interventions, psychological therapies or memory support groups
- Case studies or series of case studies
- Non-human studies
- Full text not readily available in, or not in English
- Reviews, editorials, opinion pieces or letters to editor with no original findings.
- Non-peer-reviewed material

All exclusions with detail of the decision process will be recorded. The search findings will be presented, in summary, in a PRISMA flow diagram [40].

### Data extraction and mapping results

#### Data management and extraction

Database results will be imported to Endnote software for reference management. Duplicates will be removed by authors AB and SL as described above. Microsoft Excel will be used to record the following demographic and clinical data for each included study:

- Metadata
  - ◦ Authors
  - ◦ Publication date
- Study design
  - ◦ Design
  - ◦ Outcome measures
  - ◦ Type of analysis
  - ◦ Sampling strategy
  - ◦ Setting
  - ◦ Definition of memory strategy/aid used
  - ◦ Target component memory process (e.g. encoding, retrieval, recognition)
  - ◦ Memory aids/strategies/assistive technology/name of aid/strategy
  - ◦ Frequency, intensity and duration
  - ◦ Modality (visual/verbal)
  - ◦ Delivery (carer/clinician/patient)
- Demographics
  - ◦ Sample size
  - ◦ Age
  - ◦ Gender
  - ◦ Educational attainment
  - ◦ Cohort origin/ethnicity
- Clinical data
  - ◦ Diagnosis/type of dementia
  - ◦ Severity of dementia
  - ◦ Duration of intervention
  - ◦ Comorbidities/medication
  - ◦ Findings

### Collating, summarising and reporting results

In keeping with published scoping review guidance, we will not formally evaluate the quality of the included studies. Overall results will be tabulated and a basic numerical and descriptive summary will be developed based on the methods, interventions, population and clinical data. In keeping with the research objective, a narrative report will be developed focusing on the breadth and characteristics of the memory strategies and aids used in dementia, and the suitability for translation to people with PCA, and gaps in the utility of existing memory aids and strategies for PCA. The data will be explored, with a focus on describing the characteristics and utility of the memory strategies used, in keeping with the research objectives. Experts in PCA will be consulted to inform translational value and directions for future research.

### Dissemination and ethics

The completed review will be prepared for journal publication. Preliminary findings will be presented at relevant conferences. Ethical approval is not required for this study.

## Discussion

This scoping review will provide an overview of the memory strategies and aids used in people with AD and related dementias, where memory is a primary symptom. This will allow the evaluation of the interventions and identification of specific strategies that may be transferable to a PCA population. The pilot searches have identified relevant literature and a breadth of literature covering both internal and external types of memory aids and strategies. We successfully captured recent studies on assistive technologies and digital aids, as well as more traditional memory aids and strategies, such as mnemonics and diaries. We are confident, therefore, that the full scoping review will yield relevant information, although those that may be applicable to the PCA population remain to be explored. Identifying these strategies and their strengths and limitations for a PCA population will help direct the adaptation and development of memory aids for this patient group to support post-diagnostic management.

## Supplementary Information

**Additional file 1.** Search Strategy used for PsycINFO. Scoping review search strategy.

## Data Availability

No dataset will be produced or analysed for this study; therefore, data sharing is not applicable. The search strategy for PsycINFO is available in Additional file 1.

## Abbreviations

PCA: Posterior cortical atrophy
AD: Alzheimer’s disease
AT: Assistive technologies
MCI: Mild cognitive impairment
CINAHL: Cumulative Index to Nursing and Allied Health Literature
PRISMA: Preferred Reporting Items for Systematic Reviews and Meta-Analyses
