# Supplementary material for: Suitability of memory aids and strategies for people with posterior cortical atrophy: protocol for a scoping review
Source: Syst Rev. 2023 Mar 30;12:58. doi: 10.1186/s13643-023-02187-4 (PMC10061751; doi:10.1186/s13643-023-02187-4)
Supplement: Supplementary file 1 — Additional file 1. Search Strategy used for PsycINFO. Scoping review search strategy. [file 13643_2023_2187_MOESM1_ESM.pdf]

Search Strategy for PsycINFO search; scoping review

1. (dementia).ti,ab
2. *DEMENTIA/
3. (alzheimer*).ti,ab
4. *"ALZHEIMER'S DISEASE"/
5. ("posterior cortical atrophy").ti,ab
6. (PCA).ti,ab
7. ("mild cognitive impairment*").ti,ab
8. (1 OR 2 OR 3 OR 4 OR 5 OR 6 OR 7)
9. (memory).ti,ab
10. (memories).ti,ab
11. *MEMORY/
12. (9 OR 10 OR 11)
13. ("memory strateg*").ti,ab
14. ("memory aid*").ti,ab
15. (compensatory).ti,ab
16. (restorative).ti,ab
17. ("enhanced learning").ti,ab
18. (external ADJ2 strateg*).ti,ab
19. (internal ADJ2 strateg*).ti,ab
20. (internal ADJ2 aid*).ti,ab
21. (external ADJ2 aid*).ti,ab
22. ("assistive technolog*").ti,ab
23. ("assistive device*").ti,ab
24. ("task modificat*").ti,ab
25. ("task adaptat*").ti,ab

26. ("environment* modificat*").ti,ab

27. ("environment* adaptat*").ti,ab

28. (remind*).ti,ab

29. (Plann*).ti,ab

30. (calendar*).ti,ab

31. (sign*).ti,ab

32. (list*).ti,ab

33. (checklist*).ti,ab

34. (diar*).ti,ab

35. (mental picture*).ti,ab

36. ("expand* rehearsal*").ti,ab

37. ("space* retrieval*").ti,ab

38. (cue*).ti,ab

39. (prompt*).ti,ab

40. (chunking).ti,ab

41. ("method of loci").ti,ab

42. (mnemonic*).ti,ab

43. ("action learn*").ti,ab

44. ("action based learn*").ti,ab

45. ("errorless learn*").ti,ab

46. (chaining).ti,ab

47. (modelling).ti,ab

48. (modeling).ti,ab

49. ("semantic associat*").ti,ab

50. ("memory navigat*").ti,ab

51. ("memory device*").ti,ab

52. ("adaptive technolog*").ti,ab

53. ("adaptive device*").ti,ab

54. ("prompting tool*").ti,ab

55. ("prompting device*").ti,ab

56. ("electronic* device*").ti,ab

57. ("electronic* aid*").ti,ab

58. (smartphone*).ti,ab

59. (tablet*).ti,ab

60. (app*).ti,ab

61. (13 OR OR 14 OR 15 OR 16 OR 17 OR 18 OR 19 OR 20 OR 21 OR 22 OR 23 OR 24 OR 25 OR 26 OR 27 OR 28 OR 29 OR 30 OR 31 OR 32 OR 33 OR 34 OR 35 OR 36 OR 37 OR 38 OR 39 OR 40 OR 41 OR 42 OR 43 OR 44 OR 45 OR 46 OR 47 OR 48 OR 49 OR 50 OR 51 OR 52 OR 53 OR 54 OR 55 OR 56 OR 57 OR 58 OR 59 OR 60)

62. 8 AND 12 AND 67 [DT 1990-2020] [Languages English]
